# Nativeness-constrained diffusion framework for nanobody design

**DOI:** 10.1093/bib/bbaf631.049

**Published:** 2025-12-12

**Authors:** Yinghan Zhang, Tao Jiang, Cheuk Shuen Li

**Affiliations:** Department of Surgery, The Chinese University of Hong Kong, Hong Kong; Department of Surgery, The Chinese University of Hong Kong, Hong Kong; Saint Novel Biotech Limited, Hong Kong; University of Hong Kong, Hong Kong

## Abstract

**Aim:**

Nanobodies are single-domain antibodies with promising therapeutic potential, yet accurate complementarity-determining region (CDR) design remains challenging.

**Methods:**

Existing diffusion-based frameworks such as DiffAb^[1]^ and RFantibody^[2]^ typically generate CDR sequences on fixed scaffolds by enforcing structural and physical constraints, but they neglect evolutionary information intrinsic to antibody repertoires. We propose a scaffold-constrained diffusion framework that integrates a nativeness prior, guiding generation toward sequences that are not only structurally consistent but also evolutionarily plausible.

**Results:**

By incorporating nativeness-aware constraints during design, our model produces nanobody candidates that better balance physical compatibility with natural repertoire characteristics, achieving improved sequence realism and developability.

**Conclusion:**

This approach provides a new computational strategy for antibody engineering, enabling more realistic and efficient nanobody design.

**References:**

1. Luo S., Su Y., Peng X., Wang S., Peng J., Ma J. ‘**Antigen-specific antibody design and optimization with diffusion-based generative models for protein structures.**’ Advances in Neural Information Processing Systems 2022.

2. Bennett N.R., Watson J.L., Ragotte R.J., Borst A.J., See D.L., Weidle C., Biswas R., Shrock E.L., Leung P.J.Y., Huang B., Goreshnik I., Ault R., Carr K.D., Singer B., Criswell C., Vafeados D., Garcia Sanchez M., Kim H.M., Vázquez Torres S., Chan S., Baker D. ‘**Atomically accurate de novo design of single-domain antibodies.**’ bioRxiv 2024.

